# The Non-Coding Landscape of Cutaneous Malignant Melanoma: A Possible Route to Efficient Targeted Therapy

**DOI:** 10.3390/cancers12113378

**Published:** 2020-11-15

**Authors:** Andreea D. Lazăr, Sorina Dinescu, Marieta Costache

**Affiliations:** 1 Department of Biochemistry and Molecular Biology, University of Bucharest, 050095 Bucharest, Romania; andreea.lazar@bio.unibuc.ro (A.D.L.); marieta.costache@bio.unibuc.ro (M.C.); 2 Research Institute of the University of Bucharest, 050663 Bucharest, Romania

**Keywords:** cutaneous melanoma, invasion, metastasis, miRNA, lncRNA, biomarkers, drug resistance, targeted therapy, immunotherapy

## Abstract

**Simple Summary:**

Despite improvements in cancer therapy, cutaneous malignant melanoma often remains unresponsive or quickly acquires drug resistance, proving fatal in advanced stages. Studies have shown that non-coding RNA molecules play a role in treatment-resistance. In this paper, we summarised the impact of miRNAs and lncRNAs on melanoma invasion and metastasis, pointing out their interference with BRAF inhibitors and immunotherapy. Important candidates include miR-28, miR-100, miR-125a, miR-125b, miR-200c, miR-211, SAMMSON, MELOE and HOTAIR. We also highlighted the potential of ncRNAs as promising biomarkers and molecular therapeutic targets for prospective clinical applications.

**Abstract:**

Considered to be highly lethal if not diagnosed in early stages, cutaneous malignant melanoma is among the most aggressive and treatment-resistant human cancers, and its incidence continues to rise, largely due to ultraviolet radiation exposure, which is the main carcinogenic factor. Over the years, researchers have started to unveil the molecular mechanisms by which malignant melanoma can be triggered and sustained, in order to establish specific, reliable biomarkers that could aid the prognosis and diagnosis of this fatal disease, and serve as targets for development of novel efficient therapies. The high mutational burden and heterogeneous nature of melanoma shifted the main focus from the genetic landscape to epigenetic and epitranscriptomic modifications, aiming at elucidating the role of non-coding RNA molecules in the fine tuning of melanoma progression. Here we review the contribution of microRNAs and lncRNAs to melanoma invasion, metastasis and acquired drug resistance, highlighting their potential for clinical applications as biomarkers and therapeutic targets.

## 1. Introduction

Malignant melanoma (MM) is considered one of the most aggressive and treatment-resistant human cancers, frequently leading to metastasis and accounting for the majority of skin cancer-related deaths worldwide [1,2]. Melanoma arises from the uncontrolled proliferation of melanocytes, a typically low proliferative cell population derived from neural crest progenitors [3]. Exposure to ultraviolet (UV) radiation induces the proliferation of melanocytes and the synthesis of melanin, the photoprotective pigment that is distributed to neighbouring keratinocytes via large specific vesicles termed melanosomes. Prolonged and intense exposure to UV is thought to be the main factor that determines melanomagenesis, as UV radiations can trigger oxidative stress and induce point mutations haphazardly across the genome, which can lead to loss of cell cycle control and apoptosis escape [4,5]. Extensive data from The Cancer Genome Atlas (TCGA) project partly defined the complex genetic landscape of melanoma, disclosing the high occurrence of somatic mutations and the prevalent activation of the mitogen-activated protein kinase (MAPK) pathway [6].

Classically, melanoma onset and progression has been depicted using the Clark model, which is based on a stepwise evolution of morphological abnormalities accompanying cancer. This model describes the linear progression of normal melanocytes through various precursor stages that ultimately lead to metastatic melanoma [7,8]. Benign nevus formation, followed by development of dysplastic phenotype, represent the primary events from which melanoma progression can occur. This stage is characterized by the disruption of p16^INK4a^-retinoblastoma (Rb) cascade, mostly due to *CDKN2A* mutation and subsequent inactivation [9]. Continuous and uncontrolled cell proliferation permits the protrusion of abnormal melanocytes into the epidermal/dermal junction and within the dermis in a horizontal manner, establishing the radial growth phase (RGP), at which point cells exhibit an immortal phenotype, due to the activation of human telomerase reverse transcriptase (hTERT) [10]. Loss of E-cadherin, coupled with the aberrant expression of N-cadherin and αVβ3 integrin seem to be crucial for the progression of melanoma to the final cutaneous stage, characterised by a vertical growth phase (VGP) into the dermis, where neoplastic melanocytes suffer mutations repressing apoptosis, in order to survive in the absence of keratinocytes [11,12]. Further mutations lead to the upregulation of several proteins that fuel the activity of various signalling pathways involved in cell proliferation and survival, such as loss of PTEN, RAS over-expression and β-catenin activation [1,13]. Additionally, once present in the dermal layer, melanoma cells can interact with other cell types (e.g., fibroblasts, immune cells, etc.) and gain access to blood and lymphatic vessels, facilitating their potential future invasion [7,8]. In short, the linear progression model holds that metastasis ensues later in the process of tumorigenesis, from relatively rare disseminating clones with metastatic competency, only after all previous steps have been completed [14,15]. The accumulation of genetic changes is thought to drive transition through these stages [12]. The Clark classification is still used for melanoma stadiation, and although it likely depicts the evolution of some melanomas, observations from several pathologic, epidemiologic, and experimental studies, including comparative sequencing of primary tumours and metastases, argue in favour of a less linear and more complex progression model for melanoma [16,17,18]. Substantial evidence favours the parallel progression model, akin to metastatic dormancy, which suggests that MM can originate from each of the previously described phases, without necessarily passing through all of them, as dissemination of precancerous or malignant cells to distant sites often occurs from early transformed lesions, and acquisition of important genetic alterations is not exclusively confined to the primary lesion, but rather takes place at the metastatic sites [8,17,19,20,21].

Regardless of the dilemma posed by melanoma progression, frequent functional mutations have been found and used as molecular targets to develop specific inhibitors. Considering that around half of melanomas harbour mutations in *BRAF* V600E, the first targeted therapies approved by the U.S. Food and Drug Administration (FDA) were vemurafenib and dabrafenib, followed by BRAF/MEK inhibitor pairing treatment [22]. Although they may initially provide remarkable tumour regression, resistance to therapy occurs within a few months [23,24]. So far, the most promising therapy makes use of immune checkpoint blockers (anti-PD-1 and anti-CTLA-4) that restore the function of cytotoxic T cells, thereby reactivating immune recognition and elimination of melanoma cells [25,26]. Despite a significantly prolonged progression-free and overall survival, most patients treated with immune inhibitors do not have a durable response and develop resistance within a year [27].

Non-coding RNAs (ncRNAs) have been observed to tamper with the efficiency of molecular therapies, in addition to facilitating melanoma invasion and metastasis [28,29]. They represent important epigenetic and epitranscriptomic regulators, classified and differentiated into long (lncRNAs) and small (sncRNAs) molecules, according to their length which can be longer or smaller than 200 bps. While multiple types of RNAs can be found in the small category, particular interest has been devoted to studying the deregulation of microRNAs (miRNAs/miR) in melanoma [30,31]. miRNAs can modulate the gene expression at post-transcriptional level mostly by interfering with the 3′UTR region of mRNAs, and substantial evidence points to their involvement in each stage of melanoma progression [28,31]. In contrast, the epigenetic control exerted by lncRNAs depends on the recruitment of regulatory proteins at specific DNA target regions, to silence or activate gene promoters, and their role in malignant melanoma is more elusive [32,33]. Some lncRNAs have been described to stand as decoys or sequesters of miRNAs, while others play a role in stabilizing the translational ribosomal machinery [34].

In this paper, we review the contribution of miRNAs and lncRNAs in melanoma invasion and metastasis, pointing out the interplay with the tumour microenvironment; we describe their role in acquired resistance to targeted therapy and immunotherapy, and ultimately highlight their potential as promising biomarkers and molecular targets for clinical applications.

## 2. MicroRNAs Modulate Melanoma Invasion and Metastasis

**Figure 1:**
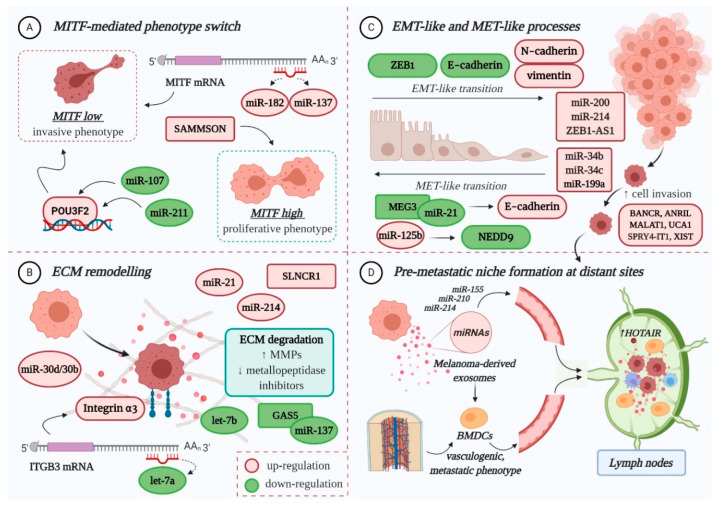
The role of non-coding RNAs in melanoma invasion and metastasis: (**A**) Both miRNAs and lncRNAs modulate the expression of MITF-M, directly or indirectly, therefore influencing the switch towards an invasive or proliferative phenotype; (**B**) The deregulated expression of ncRNAs facilitates ECM remodelling, by targeting integrins, increasing matrix metalloproteinases (MMPs) and diminishing the metallopeptidase inhibitors (TIMPs), therefore promoting ECM degradation and decreasing tumour cell-matrix adhesion; (**C**) Multiple miRNAs and lncRNAs favour the EMT-*like* process of melanoma cells, which enhances their migratory capacity, mainly by affecting the expression of specific adhesion molecules. Some ncRNAs also promote the MET-*like* process of malignant melanocytes, which leads to enhanced proliferation and colony formation, thus favouring metastasis; (**D**) The formation of a pre-metastatic niche at distant sites is mainly influenced by melanoma-derived exosomes, enriched in miRNA cargo (e.g., miR-155, miR-210, miR-214). In sentinel lymph nodes, they instigate microenvironmental changes that facilitate the recruitment, trapping and growth of circulating tumour cells. They also prime bone-marrow-derived cells (BMDCs), which achieve a vasculogenic, metastatic phenotype, and facilitate their recruitment to distant sites, where BMDCs will contribute to the formation of a pre-metastatic niche. LncRNA HOTAIR is enriched in lymph node metastases compared to primary tumours, which may suggest a role in the pre-metastatic niche formation. POU3F2: POU-domain class 3 transcription factor 2; NEDD9: Neural precursor cell expressed developmentally down-regulated protein 9; ZEB1: zinc finger E-box-binding homeobox 1. Colour code: red for upregulation and green for downregulation. Figure created with BioRender.com.

The role of miRNAs in melanoma progression is very tangled, therefore excellent reviews can be consulted here: [28,35,36,37]. In principle, miRNAs influence the evolution of melanoma to secondary sites mainly by: (A) regulating the expression of *MITF-M*, an essential melanocyte-and melanoma-specific transcription factor that operates as a “switch” in the establishment of an invasive or proliferative phenotype; (B) remodelling the extracellular matrix (ECM); (C) promoting epithelial-to-mesenchymal transition (EMT) and its reverse, mesenchymal-to-epithelial transition (MET); and (D) preparing the formation of the pre-metastatic niche [28,36,38,39,40] (Figure 1).

Reduced expression of *MITF-M* in melanoma cells determines the acquisition of an invasive phenotype [39]. Several miRNAs have been found to regulate the activity of this lineage-restricted gene at a post-transcriptional level, among them miR-182, miR-137, miR-211 and miR-107 [40,41,42,43,44]. Overexpression of miR-182 stimulates melanoma cell migration and invasion through the direct downregulation of MITF and FOXO3 expression; studies on melanoma cell lines and tissue samples have found that its expression increases gradually from primary to metastatic stage [42]. Similarly, high levels of miR-137 expression have been correlated with poor survival in advanced melanoma patients [45]. In contrast, decreased expression of miR-211 can be observed in highly invasive melanoma cell lines [46]. Normally, miR-211 prevents the loss of cell adhesion through the negative modulation of NUAK1 [47], thereby inhibiting the migratory and invasive capacity of melanoma cells [48]. Additionally, it has been proposed that miR-211 can transcriptionally repress POU3F2 (POU-domain class 3 transcription factor 2), also known as BRN2 (brain-specific homeobox 2), which is a known suppressor of *MITF*. As such, loss of miR-211 can increase the expression of BRN2, and therefore inhibit *MITF*, ensuring that malignant melanocytes are kept in a pro-invasive, dedifferentiated state [43]. Recently, Zhao et al. reported the downregulation of miR-107, another tumour suppressor that inhibits melanoma cell invasion by targeting POU3F2 [44]. In BRAF-mutant cells, overexpression of BRN2 contributes to cytoskeletal rearrangement and increased cell invasion [49].

Several miRNAs play a role in ECM remodelling [50]. In melanoma, loss of let-7a, which is a negative regulator of *ITGB3*, leads to elevated levels of integrin α3 [51], while suppression of let-7b indirectly enhances the production of matrix metalloproteinase (MMP)-9 [52], facilitating collagen degradation and cell invasion. Meanwhile, miR-21 is thought to induce an invasive phenotype in melanoma cells by targeting the mRNA of tissue inhibitor of metalloproteinases (TIMP)-3 [53]. Lastly, the increased expression of miR-30d/miR-30b cluster stimulates the invasive and metastatic potential of melanoma cells, both in vitro and in vivo, potentially by silencing GALNT7 (polypeptide N-acetylgalactosaminyltransferase 7), which strongly affects the O-glycosylation of membrane proteins, and subsequent interaction with the ECM [54].

True EMT in melanoma is not possible as melanocytes are not epithelial cells, however some miRNAs are implicated in an EMT-like process that promotes the invasion and metastasis of tumour cells. One of them is the cluster miR-224/miR-452, which silences a metastatic suppressor that normally inhibits E2F1, and which has been shown to facilitate the cytoskeletal rearrangement of less aggressive cells, resulting in increased migratory and invasive propensity [55]. Also considered of particular relevance, the miR-200 family (miR-200a, miR-200b, miR-200c, and miR-141) drives the EMT-like process by upregulating the expression of Bmi-1 oncogene, which in turn promotes the activation of PI3K/AKT and MAPK cascades. This negatively influences the expression of ZEB1 (zinc finger E-box-binding homeobox 1) and E-Cadherin, at the same time stimulating N-Cadherin and vimentin expression [56]. High levels of miR-214 have also been associated with enhanced cell motility and metastatic potential, as this specific miRNA indirectly downregulates the expression of some adhesion molecules (MCAM, E-cadherin) and metallopeptidase inhibitors (TIMP1, TIMP2) [57].

The MET-like process of malignant melanocytes is favoured by miR-125b overexpression, who directly targets a transcript of NEDD9 (neural precursor cell expressed developmentally down-regulated protein 9) [58,59], and whose in vitro inhibition was found to decrease the invasive potential of aggressive melanoma cells [58]. Other miRNAs (miR-34b, miR-34c, and miR-199a-3p) also contribute to this mesenchymal movement by targeting the mRNA of tyrosine-protein kinase Met (c-MET), whose increased expression facilitates melanoma cell migration and metastasis [60,61].

miRNAs also participate in the formation of a pre-metastatic niche at distant organs that will enable the implantation and survival of tumour cells. Melanoma-derived exosomes, with their enriched miRNA cargo (e.g., miR-155, miR-210, miR-214), are primarily involved in this intercellular communication (for review: [62,63]). For instance, exosomes recruited into sentinel lymph nodes promote the upregulation of proteases that degrade the ECM and enhance the expression of pro-angiogenic factors (TNFα, VEGF, etc.) to facilitate the recruitment, trapping and growth of malignant melanocytes within the metastatic niche [64,65]. They are also capable of priming bone-marrow-derived cells (BMDCs) to achieve a vasculogenic, metastatic phenotype, and facilitate their recruitment to metastatic sites where they will contribute to the establishment of a suitable microenvironment for trapping circulating melanoma cells [66].

## 3. LncRNAs Modulate Melanoma Invasion and Metastasis

Despite the emerging interest and growing evidence of the contribution of lncRNAs in cancer [67,68], little is known about the impact of deregulated expression of lncRNAs in the invasion and metastasis of MM [29,69] (Figure 1).

The first lncRNA characterized was SPRY4-IT1, a transcript derived from an intron of the *SPRY4* gene, whose expression is increased in melanoma [70]. It was identified as a regulator of several processes, as suppression of SPRY4-IT1 resulted in abnormal cell growth, differentiation and apoptosis, as well as decreased invasion capacity of melanoma cell lines [70,71]. Although the molecular mechanisms that interfere with the invasion of MM are not clear, a study concerning non-small cell lung carcinoma revealed a possible role in the activation of EMT by modulating both E-cadherin and vimentin expression, leading to cell proliferation and metastasis [72]. Interestingly, another lncRNA with a potential role in regulating EMT was discovered by Siena et al., while exploring the lncRNA gene expression patterns across melanocytes, primary and metastatic melanoma cells. They found a significant upregulation of ZEB1 antisense RNA 1 (ZEB1-AS1) in melanoma cells. Data analysis from TCGA confirmed the increased expression of *ZEB1-AS1* in metastatic melanoma and its association with hotspot mutations in *BRAF* and *RAS* family genes. Additionally, enrichment analysis correlated *ZEB1-AS1* with the gene expression of zinc finger E-box binding homeobox 1 (*ZEB1*), an essential EMT marker, suggesting a possible role in melanoma invasiveness and phenotype switching [73].

TCGA data analysis also confirmed the clinical relevance of SRA-like non-coding RNA1 (SLNCR1), a lncRNA whose highly conserved sequence is strikingly similar to that of lncRNA steroid receptor RNA activator 1 (SRA1), and whose increased expression is associated with shorter overall survival in melanoma patients. Functional and mechanistic studies revealed SLNCR1 promotes melanoma invasion through upregulation of MMP9 (involved in ECM degradation) in cooperation with the brain-specific homeobox protein 3a (Brn3a) and the androgen receptor (AR) [74].

A screen of differentially expressed lncRNAs in *BRAF* V600E mutated melanoma cells has led to the identification of BRAF-activated non-coding RNA (BANCR) as a putative regulator of cell proliferation and motility. Overexpression of BANCR in MM promotes the activation of the extracellular signal-regulated kinases 1/2 (ERK1/2) and c-Jun N-terminal kinase (JNK) components of the MAPK pathway, while its knockdown affects the migratory capacity of tumour cells [75,76]. A positive feedback mechanism with the V600E mutation is thought to induce BANCR overexpression, subsequent upregulation of chemokines and increase in melanoma cell motility [76,77].

The oncogenic activity of antisense non-coding RNA in the INK4A locus (ANRIL) in melanoma revolves around the regulation of its encoding locus that also harbours the tumour suppressor genes *INK4A* and *INK4B.* In cutaneous melanoma tissue samples and cell lines, strong levels of ANRIL negatively modulated the expression of CDKN2A/2B proteins [78,79]. In addition, knockdown experiments managed to restore *INK4A* and *INK4B* expression, at the same time suppressing in vitro colony formation and migration [80]. Meanwhile, the encoding gene of survival associated mitochondrial melanoma-specific oncogenic lncRNA (SAMMSON) also harbours the melanoma-specific oncogene *MITF* and it was demonstrated that SAMMSON is frequently co-amplified with it [81]. As shown by functional assays, knockdown of this lineage-specific lncRNA drastically affects cell proliferation and viability, even sensitizing melanoma to MAPK-targeted drugs. Mechanistically, SAMMSON interacts with mitochondrial protein p32, a critical regulator of tumour metabolism [81].

Although the molecular mechanisms are currently unknown, HOX transcript antisense intergenic RNA (HOTAIR) is perceived as a regulator of melanoma invasion and metastatic progression, considering it was found particularly enriched in lymph-node metastases compared to primary lesions and knockdown of HOTAIR in vitro suppressed melanoma cell motility and invasion [82]. In a similar manner, a significantly higher expression of metastasis-associated lung adenocarcinoma transcript 1 (MALAT1) and urothelial carcinoma associated 1 (UCA1) was observed in advanced stages of MM, than in early stages, suggesting the potential role of MALAT1 and UCA1 in melanoma invasion and metastasis [83]. Moreover, a subsequent mechanistic study demonstrated that UCA1 can interact with miR-507 and promote the inhibition of FOXM1 expression, leading to increased invasiveness and clonogenic potential of melanoma cells [84].

Recently, the upregulated expression of X-inactive-specific transcript (XIST) in MM tissues and resistant cell lines was reported, with XIST being proposed as a crucial regulator in melanoma progression [85]. Through the use of bioinformatics, XIST was revealed to act as a molecular sponge for miR-21, which targets PI3KR1, a regulatory subunit of PI3K. Functional studies concerning XIST repressed PI3KR1 and AKT expression, leading to inhibition of melanoma cell proliferation and migration, at the same time increasing sensitivity to oxaliplatin [85].

Lastly, lncRNAs can also act as tumour suppressors in melanoma, however only growth arrest-specific transcript 5 (GAS5) and maternally expressed gene 3 (MEG3) have been reported so far [86,87]. Lentiviral-mediated overexpression of GAS5 diminished the expression of MMP2, a specific protein involved in ECM degradation, and reduced the migratory and invasive capacity of human MM cells [88]. Interestingly, GAS5 seems to repress melanoma tumorigenesis via miR-137, while MEG3 inhibits tumour growth and metastasis by modulating miR-21/E-cadherin axis [87,89].

## 4. Non-Coding RNAs and the Interplay with the Melanoma Microenvironment

**Figure 2:**
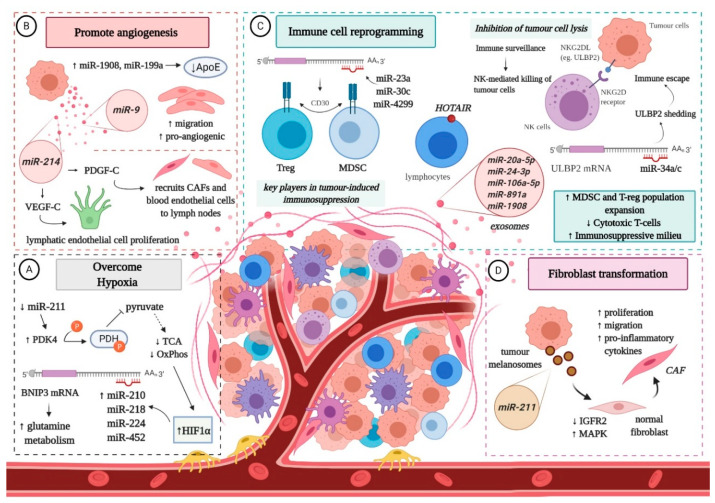
The interplay of non-coding RNAs with the melanoma microenvironment: (**A**) Melanoma cells overcome oxygen deprivation. Downregulation of miR-211 elevates PDK4 (pyruvate dehydrogenase kinase 4) levels, leading to the inhibition of PDH (pyruvate dehydrogenase) via phosphorylation, which in turn limits oxidative phosphorylation as pyruvate is rerouted away from the TCA (tricarboxylic acid cycle). Increased levels of HIF1α (hypoxia-inducible factor 1 alpha) lead to the upregulation of several miRNAs that target BNIP3 (BCL2/adenovirus E1B interacting protein 3), stimulating glutamine metabolism; (**B**) MicroRNAs promote neo-angiogenesis. Certain miRNAs are upregulated in melanoma cells and distributed to endothelial cells via exosomes, in order to increase the migratory and pro-angiogenic potential of said cells and recruit them to the MME, found either in the cutaneous tissue or in lymph nodes; (**C**) Malignant melanocytes reprogramme immune cells to favour the establishment of an immunosuppressive microenvironment. In principle, miRNAs (often transported via exosomes) recruit and promote the expansion of MDSCs (myeloid-derived suppressor cells) and T-regs (regulatory T cells), inhibit cytotoxic T cells and trick NK (natural killer) cells by shedding soluble ligands in order to avoid tumour cell lysis. The role of HOTAIR in this case is uncertain, even though its overexpression was observed in the plasma membrane of lymphocytes associated with the MME; (**D**) Melanoma-derived melanosomes deliver miR-211 to normal fibroblasts, which inhibits the expression of IGFR2 (insulin-like growth factor receptor 2) and activates MAPK signalling, to transform them into cancer-associated fibroblasts (CAF) and prepare the dermis for future invasion. ApoE: Apolipoprotein E; PDGF-C: Platelet-derived growth factor C; VEGF-C: Vascular endothelial growth factor C; NKG2D: natural killer group 2, member D; ULBP2: UL16 binding protein 2. Figure created with BioRender.com.

The tumour microenvironment (TME) represents one of the hallmarks of cancer invasion and metastasis. The crosstalk between melanoma cells and the complex network composed of soluble factors, ECM and different types of cells, such as endothelial cells, fibroblasts and immune cells, ensures malignant progression to eventual metastasis [28]. Specific ncRNAs, especially miRNAs, can regulate the dynamic of melanoma microenvironment (MME), playing a role in: (A) surviving hypoxia; (B) angiogenesis; (C) immune cell reprogramming; and (D) conversion of normal fibroblasts into cancer-associated fibroblasts (CAFs) [90,91] (Figure 2).

Hypoxic conditions promote the invasive potential of malignant melanocytes [92] and increase the amount of specific miRNAs in exosomes (e.g., miR-494, miR-513a, miR-4497, miR-6087) [93,94]. Hypoxia-inducible factor 1 alpha (HIF1α), key player in the cellular response to oxygen deprivation [95], promotes the expression of several miRNAs (miR-210, miR-218, miR-224, and miR-452) that contribute to tumour cell plasticity and aggressiveness, by targeting and increasing *BNIP3* (BCL2/adenovirus E1B interacting protein 3), associated with glutamine metabolism [96,97,98]. In addition, low expression of miR-211 leads to the upregulation of the metabolic regulator PDK4, which triggers a decrease in pyruvate dehydrogenase and subsequent oxidative phosphorylation [99].

Another mechanism that favours melanoma cell proliferation and survival in oxygen deprived environments is neo-angiogenesis [100]. It has been reported that miR-1908 and miR-199a negatively regulate the expression of ApoE, a known suppressor of angiogenesis and cell invasion [60,101]. Moreover, melanoma cells with high levels of miR-1908 and miR-199a are more capable of recruiting endothelial cells in vitro and in vivo [60]. Furthermore, the transfer of miR-9 from malignant melanocytes to endothelial cells via exosomes stimulates their migratory and pro-angiogenic potential through the activation of the JAK-STAT pathway [102]. Meanwhile, miR-214 is associated with increased expression of VEGF-C and PDGF-C, which facilitate angiogenesis in lymph nodes, either by inducing the proliferation of lymphatic endothelial cells, or by serving as a chemoattractant for CAFs and blood endothelial cells [103,104].

Melanoma-derived miRNAs closely interact with immune cells to facilitate the escape of tumour cells from immune surveillance, and thus contribute to melanoma progression [105]. For instance, miR-210 impairs the cytotoxic activity of T cells by down-regulating TNF-α, IL-6, and IFN-β [106], while miR-30c, miR-23a and miR-4299 selectively modulate the expression of CD30 in regulatory T cells (T-regs) and myeloid-derived suppressor cells (MDSCs), facilitating the development of an immunosuppressive milieu [28]. Additionally, high expression of miR-30d, coupled with low levels of GALNT7, stimulates the secretion of IL-10, reducing the activation and recruitment of immune cells. Interestingly, overexpression of miR-30d is also associated with an upsurge of FOXP3-positive lymphocytes, diminution of CD3+ T cells recruitment and induction of T-regs [54]. Furthermore, by targeting *suppressor of cytokine signalling 1 (SOCS1)*, miR-155 is able to promote the recruitment of MDSCs to the MME [107]. On top of that, the exosomal transfer of miR-20a-5p, miR-24-3p, miR-106a-5p, miR-891a and miR-1908 stimulates expansion of T-regs and MDSCs, at the same time promoting the Th1-polarization of CD4+ cells [108].

Natural Killer (NK) cells play an important role in cancer immune surveillance through the interaction between the NKG2D (natural killer group 2, member D) receptor and its ligands (NKG2DL), which enables them to identify and destroy tumour cells [109]. However, malignant melanocytes can slip under the radar of NK cells due to miR-34a/miR-34c overexpression [110]. The cluster binds the 3′UTR region of ULBP2 (UL16 binding protein 2), a stress-induced ligand of NKG2D, promoting immune escape by enhancing its shedding from melanoma cells [110], which are detectable in serum samples from melanoma patients [111].

Although most studies have focused on the miRNA cargo of exosomes and their influence on the MME, a new concept has been put forth: the dermal niche formation for prospective invasion mediated by miRNAs transported via melanosomes, a melanocyte-specific class of large vesicles (~500 nm) originated from the endosomal system [91]. The role of melanosomes in melanoma progression has been questioned because neoplastic melanocytes retain their ability to synthesize and transfer melanosomes to nearby cells. Dror et al. discovered that melanoma melanosomes reprogram dermal fibroblasts into CAFs, capable of increased cell proliferation, migration and secretion of pro-inflammatory cytokines. The molecular mechanism behind this transformation seems to be associated with the transfer of miR-211, which targets and silences the tumour suppressor IGFR2 (insulin-like growth factor receptor 2), whose downregulation enhances the activity of MAPK signalling pathway. These findings suggest that melanoma cells shape the stromal niche early in the disease by manipulating dermal fibroblasts [91]. Studies on other tumour cell-related vesicles, specifically exosomes, have revealed that they enter the lymphatic vessels and accumulate in sentinel lymph nodes, through interaction with lymphocytes, where they promote cell proliferation, ECM remodelling, angiogenesis and pro-tumorigenic humoral immunity, which facilitates the subsequent trapping and growth of melanoma cells [64,65,112,113]. The role of melanosomes in this context is as yet unknown.

Hardly any data is available for the influence of lncRNAs on the MME. In this regard, aberrant expression of HOTAIR was detected in lymphocytes surrounding metastatic tumour cells in melanoma patients [114]. The study highlighted the enriched expression of HOTAIR on the plasma membrane of immune cells within TME, which would appear to be associated with specific “vesicle-like” membrane projections [115]. Even so, it is unclear whether the presence of this lncRNA on the surface of intra-tumour lymphocytes is associated with endogenous production or it represents the outcome of a signal transmission between melanoma cells and the cells of TME [116].

## 5. Non-Coding RNAs in Drug Resistance

Continuous treatment with target-based therapies remains unsuccessful and leads to melanoma relapse due to acquisition of drug resistance. Even though BRAF monotherapy (BRAFi), or its combination with MEK inhibitors (MAPKi) instigate a profound regression in patients with BRAF-mutated metastatic melanoma, their effect is only temporary [23,24]. The same issue arises with the use of immunotherapy [27]. The role of ncRNAs in the development of melanoma drug resistance has been questioned and while the contribution of miRNAs is well studied [37], the role of lncRNAs in such resistance is still largely unknown.

### 5.1. Resistance to BRAF or MAPK Inhibitors

The deregulated expression of several miRNAs, namely upregulation of onco-miRNAs and downregulation of oncosuppressors, has been shown to contribute to the acquisition of drug resistance to BRAFi or MAPKi-based therapy in melanoma (Table 1). For example, overexpression of miR-31a, miR-100 and miR-125b stimulates tumour cell proliferation and apoptosis escape, decreasing drug sensitivity in patients treated with vemurafenib. Their expression is associated with the chemokine monocyte chemoattractant protein-1 (CCL2), which promotes melanoma progression in BRAFi-resistant cells [117]. Inhibition of miR-125a leads to the partial drug resensitization of melanoma in a subset of BRAFi-resistant cell lines [118]. Also, it has been concluded that miR-204 and miR-211 facilitate the emergence of resistance to vemurafenib, as higher expression levels were found in resistant melanoma cells compared to their drug-naïve counterparts [119,120]. Another miRNA involved in the modulation of BRAFi sensitivity is miR-514a, which reportedly inhibits the tumour suppressor NF1, resulting in increased survival to therapy [121].

Upregulation of oncosuppressor miR-7 negatively impacts the expression of target genes *EGFR*, *IGF-1R* and *CRAF* in vemurafenib-resistant cells of in vitro melanoma models or xenograft mice, which in turn inhibits the activation of MAPK and PI3K/AKT pathways, in this manner reversing melanoma cell resistance to BRAFi [122]. Another oncosuppressor miRNA that indirectly targets the previously mentioned cascades by repressing the expression of *myeloid cell leukemia 1 (MCL-1)*, and therefore potentiates sensitivity to BRAFi therapies, is miR-32. It has been speculated that inhibition of MCL-1 through miR-32 may represent an efficient therapy for melanoma, regardless of its mutational status (NRAS, BRAF or PTEN), as it was discovered to exhibit synergistic effects with vemurafenib [123]. Furthermore, miR-200c prevents the establishment of drug resistance by targeting several transcriptional repressors involved in EMT, and as such this specific miRNA is significantly downregulated in resistant melanoma cells [124]. On top of that, low levels of miR-579-3p can affect not only the BRAF/MAPK signalling pathway, but also the MDM2/p53 pathway, causing uncontrolled cell proliferation and migration, coupled with inhibition of apoptosis, thus contributing to the development of MAPKi resistance [125]. Of note, overexpression of miR-579-3p was able to prevent colony formation in cells exposed to vemurafenib and impair the growth of BRAFi-resistant melanoma cells in combination with the MEK inhibitor (MEKi) trametinib [125].

Recently, Sanlorenzo et al. identified MIRAT (MAPK Inhibitor Resistance Associated Transcript), a novel cytoplasmic lncRNA, which is significantly overexpressed in melanoma cells with acquired resistance to MAPKi, and modulates MAPK signalling by binding to the MEK scaffold protein IQGAP1 [126]. Knockdown of SAMMSON was shown to sensitize melanoma to MAPK-targeting therapeutics as well, but its underlying mechanism is unknown [81]. Meanwhile, Joung et al. performed a genome-scale activation screen and found EMICERI (EQTN MOB3B IFNK C9orf72 enhancer RNA I), a novel lncRNA that confers resistance to vemurafenib through upregulation of MOB3B and subsequent activation of the Hippo signalling pathway [127].

### 5.2. Resistance to Immunotherapy

To date, results regarding the effect of ncRNAs on melanoma response to immune checkpoint blockers are sparse [128,129]. In order for studies to gain momentum in this area, some suggested the use of single-cell RNAseq approach to distinguish between the expression of cancer cells and that of the immune components [90]. Nevertheless, some miRNAs have been recognised as being involved in the conversion of monocytes into immunosuppressive MDSCs (let-7e, miR-99b, miR-100, miR-125a, miR-125b, miR-146a, miR-146b, miR-155) [130], while some interfere with PD-1 (miR-28) or PD-L1 (miR-17-5p) at a post-transcriptional level [131,132], facilitating resistance to immunotherapy (Table 2). LncRNAs have also been identified as potential modulators of myeloid cell differentiation towards an immunosuppressive phenotype (olfr29-ps1, lnc-chop) [133,134], however it seems that one particular polycistronic lncRNA, namely *melanoma-overexpressed antigen* (MELOE), can unexpectedly improve antigen presentation after being translated into short polypeptides, potentially increasing melanoma immunotherapy efficiency [135] (Table 2). The highly specific melanoma antigens MELOE-1 and MELOE-2 are involved in T cell immunosurveillance and might prove valuable as T cell targets for immunotherapy [135]. Interestingly, adoptive transfer of tumour-infiltrating T lymphocytes able to recognize MELOE-1 contributed to the relapse-free survival of melanoma patients over an extended period of time [136].

## 6. Clinical Applications of Non-Coding RNA Molecules in Melanoma Management

The clinical utilities and implications of ncRNAs in melanoma management are not fully established and future investigations are needed to clarify this aspect [29,90], however some promising results that recommend miRNAs and lncRNAs as tools for diagnosing, monitoring and treating cutaneous melanoma are presented in the following sections.

### 6.1. Non-Coding RNAs as Circulating Biomarkers for Cutaneous Melanoma

Even though lots of therapeutic progress has been made throughout the past years in finding alternative options for melanoma prognosis, it still remains challenging to find minimal invasive methods to follow up cancer progression and metastasis. Thus, liquid biopsies rise as a useful tool for the detection of circulating cancer biomarkers, among them ncRNAs [137,138,139].

Up to date lots of effort and work has been put in identifying a landscape of miRNAs as circulating biomarkers for melanoma diagnosis and prognosis (Table 3). Among the first to address this matter, Leidinger et al. have performed high throughput methods to validate a spectrum of 16-miRNAs which could be identified only in melanoma positive patients [140]. Several studies have indicated an increased level of multiple miRNAs, such as miR-19a, miR-149 and miR-126, in the plasma of metastatic melanoma patients as compared to healthy controls, suggesting these molecules to be actively involved in melanoma progression [141,142,143]. Interestingly, the co-detection of miR-185 and miR-1246 in liquid samples could offer an accurate identification of patients with metastatic melanoma, which could allow an early cancer diagnosis [144]. Additionally, Van Laar et al. have proposed a 38-miRNA signature (MEL38) in order to designate melanoma from normal plasma samples and an 18-miRNA signature (MEL18) which has the means to differentiate non-metastatic (stage I/II) and metastatic (stage III/IV) melanoma subjects [145]. A study done by Li et al. has detected miR-221 in serums samples from cutaneous MM patients, making this molecule a potential biomarker for melanoma evolution [146]. Another 4 miRNAs molecules (miR-30d, miR-15b, miR-150 and miR-425) have been proposed as potential biomarkers in order to discriminate between low and high-risk cases of recurrence [147]. Furthermore, Stark et al. indicated a 7-miRNA panel (MELmiR-7) including miR-16, miR-509-5p, miR-4706 and miR-211-5p which could be successfully implemented in the prediction of melanoma growth and recurrence, thus having a significant impact on melanoma prognosis and diagnosis [148].

In a similar manner, lncRNA molecules have also been identified as potential circulating biomarkers for melanoma (Table 3). In this context, it has been demonstrated that plasma lncRNA SPRY4-IT is significantly higher in tumour samples as compared to healthy controls [149]. Furthermore, Tang et al. observed the overexpression of HOTAIR in melanoma serum samples in comparison to non-cancer probes [82], while Cantile et al. found a significantly higher expression of HOTAIR in serum samples taken from patients with advanced melanoma [114]. These promising reports support the fact that HOTAIR has strong implications in the carcinogenesis of melanoma, making it a potential prognostic and diagnostic marker for MM.

### 6.2. Non-Coding RNAs as Targets for Promising Therapeutic Strategies

The potential of targeting ncRNAs to develop novel anticancer therapeutic strategies or to increase the efficacy of already existing ones has been pointed out in several studies [90,150,151].

A number of ways in which miRNAs could be used have already been analysed, among which (1) synthetic miRNA mimetic agents, that could replace lost miRNA, (2) small-molecule inhibitors of miRNA, used for suppressing miRNA biogenesis or interaction with its target, (3) anti-miRNA oligomers, which are competitive inhibitors of miRNAs, leading to an upregulation of the target mRNA, or even (4) directly targeting miRNAs in the course of their transport within the tumour milieu or to other sites [152]. For example, miR-200c has been described as a potential therapeutic target for overcoming resistance to BRAFi therapy. In BRAFi-resistant cell lines and more importantly in clinical samples, low levels of miR-200c are correlated with acquired resistance. Restoration of miR-200c expression or knockdown of its molecular target favours the effect of inhibitory drugs and impairs the establishment of resistance [124] (Table 4). However, the use of miRNAs in therapy is hampered by their poor intracellular uptake, as well as rapid degradation in biological fluids. Strategies to deliver tumour-suppressive miRNAs or interfere with tumour-promoting miRNAs are still under development [153]. For instance, Fattore et al. developed lipid nanoparticles to encapsulate miR-204-5p and miR-199b-5p, either individually or in combination, and tested them on in vitro drug resistant models. Their results showed that these lipid nanoparticles loaded with oncosuppressor miRNAs are highly efficient in impairing melanoma cell proliferation and viability, affecting key signalling cascades involved in cell survival, in addition to positively influencing the efficacy of BRAF and MEK inhibitory drugs [154] (Table 4).

The involvement of lncRNAs in melanoma has promising therapeutic implications and selective knockdown of specific lncRNAs could lead to the development of reliable therapeutic strategies (Table 4). Out of the current methods available for studying lncRNAs, small interference RNA (siRNA)-dependent knockdown is used the most [155]. For instance, siRNA-mediated knockdown of SPRY4-IT1 in melanoma cell lines prevented tumour cell growth and limited invasion [70,71]. Similar results were obtained for knockdown of HOTAIR and UCA1, including inhibition of cell motility and invasive capacity [82,84]. MALAT1 knockdown was followed by a decrease in melanoma cell migration [83], whereas colony formation and metastatic ability of cancer cells were diminished in the absence of ANRIL [80]. Knockdown of SLNCR1 decreased the invasiveness of melanoma cells, although cell proliferation and motility were not affected [74]. Administration of GAS5, a tumour suppressor lncRNA, to nude mice inhibited melanoma growth, however further studies focused on the therapeutic value of GAS5 are needed [86].

The combination of a SAMMSON-specific antisense oligonucleotide with the BRAFi dabrafenib exerted a synergistic anti-tumour effect and induced apoptosis in a patient derived tumour xenograft preclinical model, whereas dabrafenib could only restrain melanoma growth (Table 4). Moreover, no significant difference was found between the toxicity levels of administrating SAMMSON-specific antisense oligonucleotide with BRAF inhibitors in comparison to administration of BRAF or MEK inhibitors alone [81].

## 7. Conclusions

Research concerning the molecular landscape of malignant melanoma has brought impressive results in terms of patients’ overall survival in metastatic disease, due to its contribution to the development of targeted-based drugs and immunotherapy. Nevertheless, acquired resistance to therapy still remains a challenge, reflecting upon the poor prognosis of a significant number of patients. In this regard, therapeutic strategies aimed to modulate ncRNAs in combination with targeted agents and/or immunotherapy may represent a more efficient solution, considering that miRNAs and lncRNAs are not only involved in melanoma invasion and metastasis, but also facilitate resistance against currently available molecular therapeutic approaches. Important candidates include miR-28, miR-100, miR-125a, miR-125b, miR-200c, miR-211, SAMMSON, MELOE and HOTAIR. The first miRNA mentioned silences an essential immune checkpoint of cytotoxic T cells (PD-1), contributing to the immune escape of malignant melanocytes and to acquired resistance to immunotherapy [131]. miR-100, miR-125a and miR-125b also impair immunotherapy by favouring myeloid cell differentiation and polarization towards an immunosuppressive phenotype [130]. Furthermore, these miRNAs decrease drug sensitivity to BRAF inhibitors [117,118], promoting tumour cell proliferation, survival and invasion [58,117]. In BRAFi-resistant melanoma cell lines, the expression of miR-200c is significantly decreased, however its restoration prevents the establishment of drug resistance by targeting several transcriptional repressors involved in EMT [124]. Another strong potential therapeutic target is miR-211, whose downregulated expression indirectly ensures the inhibition of *MITF* and subsequent highly invasive phenotype of melanoma cells [43,46]. Low levels of miR-211 also aid malignant melanocytes in overcoming hypoxia [99], while delivery of this particular miRNA by melanoma-derived melanosomes to normal fibroblasts promotes their conversion into CAFs, priming the dermis for future invasion [91]. Remarkably, higher expression of miR-211 facilitates the emergence of resistance to vemurafenib [119,120], and its presence in liquid biopsies was found to predict melanoma growth and recurrence [148], marking it not only as a therapeutic target but also as a potential diagnostic and prognostic biomarker. Concerning lncRNA candidates, knockdown of SAMMSON drastically affects tumour cell proliferation and viability, even sensitizing melanoma to dabrafenib and MAPK-targeted drugs [81]. MELOE is of particular interest for immunotherapy, considering that it can be translated into highly specific melanoma antigens (MELOE-1 and -2), that facilitate the recognition of tumour cells by cytotoxic T cells [135]. Although the role of HOTAIR in acquired drug resistance is currently unknown, it could represent a promising therapeutic target for MM, considering it was found particularly enriched in lymph-node metastases and its knockdown suppressed melanoma cell motility, significantly decreasing invasion [82]. Higher HOTAIR expression was also detected in serum samples from patients with advanced melanoma, which suggests that this specific lncRNA may also serve as a prognostic and diagnostic marker for MM [114]. Strategies to deliver tumour-suppressive ncRNAs or interfere with tumour-promoting ncRNAs are searched for and future directions for the development of innovative treatment modalities include the use of intelligent nanocarriers loaded with ncRNAs for selective gene therapy. Although miRNAs and lncRNAs seem very promising biomarkers, their translation into the clinical area requires further studies and future investigative clinical trials.

## Figures and Tables

**Table 1:** Non-coding RNAs that contribute to resistance or sensitivity to targeted therapy with BRAF or MAPK inhibitors.

Induced Effect	Overexpression of ncRNAs	Target(s)	Drugs	References
Drug resistance	miR-34a, miR-100 and miR-125b	CCL-2	Vemurafenib	[117]
	miR-125a	BAK1, MLK3		[118]
	miR-204 and miR-211	NUAK1/ARK5, IGFBP5, TGF-bRII, Slug, CHD5		[119,120]
	miR-514a	NF1		[121]
	MIRAT	IQGAP1/MAPK signalling	Trametinib (MEKi) and PLX4720 (BRAFi)	[126]
	SAMMSON	p32 mitochondrial protein	Vemurafenib and pimasertib (MEKi)	[81]
	EMICERI	MOB3B/LATS1/Hippo signalling axis	Vemurafenib	[127]
Drug sensitivity	miR-7	EGFR/IGF-1R/CRAF	Vemurafenib	[122]
	miR-32	MCL-1		[123]
	miR-200c	Bmi-1	Vemurafenib or analog PLX4720	[124]
	miR-579-3p	BRAF, MDM2	Vemurafenib and trametinib	[125]

**Table 2:** Non-coding RNAs that interfere with immunotherapy in a negative or positive manner.

Name of ncRNAs	Function	Immune Response	References
miR-28	**Blocks immune checkpoint** Silences PD-1 by binding to its 3′UTR region	Negative	[131]
miR-17-5p	**Blocks immune checkpoint ligand** Binds to PD-L1 and contributes to melanoma resistance	Negative	[132]
Let-7e, miR-99b, miR-100, miR-125a, miR-125b, miR-146a, miR-146b, miR-155	**Control of MDSC** Favours myeloid cell differentiation and polarization towards an immunosuppressive phenotype	Negative	[130]
Olfr29-ps1	**Control of MDSC** Promotes MDSCs’ differentiation and function via de m6A-modified Olfr29-ps1/miR-214-3p/MyD88 regulatory network	Negative	[133]
Lnc-chop	**Control of MDSC** Promotes the differentiation and function of MDSCs	Negative	[134]
MELOE	**Antigen presentation** Produces immunogenic antigens (MELOE-1 and -2) that are recognized by cytotoxic T cells	Positive	[135]

**Table 3:** Promising results that recommend non-coding RNAs as circulating biomarkers for potential clinical applications.

Potential Role	Non-Coding RNAs	Sample Type	References
Circulating biomarkers for prognosis and diagnosis	16 miRNA panel (among them miR-30d and miR-17)	Blood	[140]
	miR-19a miR-126 miR-149	Plasma	[141,142,143]
	miR-185 miR-1246		[144]
	18 miRNA panel (among them miR-199b-5p and let-7e)		[145]
	miR-221	Serum	[146]
	miR-15b miR-30d miR-150 miR-425		[147]
	7 miRNA panel (among them miR-16, miR-211-5p, miR-4706 and miR-509)		[148]
	SPRY4-IT	Plasma	[149]
	HOTAIR	Serum	[82,114]

**Table 4:** Promising results that recommend non-coding RNAs as therapeutic targets for potential clinical applications.

Potential Role	Functional Studies	Research Model	Therapeutic Effect(s)	References
Targets for promising therapeutic strategies	Lentiviral overexpression of miR-200c	BRAFi-resistant cell lines	Restores sensitivity to BRAFi therapies	[124]
	Lipid nanoparticles loaded with miR-204-5p and/or miR-199b-5p	In vitro drug resistant models	Impair melanoma cell proliferation and viability Positively influence the effect of MAPKi	[154]
	siRNA-mediated knockdown of SPRY4-IT1	Malignant melanoma cell lines	Prevents tumour cell growth and limits invasion	[70,71]
	siRNA-mediated knockdown of HOTAIR		Inhibits cell motility and decreases invasion	[82]
	siRNA-mediated knockdown of UCA1		Inhibits cell proliferation and invasion Induces cell cycle arrest	[84]
	siRNA-mediated knockdown of MALAT1		Impairs melanoma cell migration	[83]
	siRNA-mediated knockdown of ANRIL		Diminishes colony formation and metastatic ability	[80]
	siRNA-mediated knockdown of SLNCR1		Decreases invasiveness of melanoma cells	[74]
	Lentiviral overexpression of GAS5	In vitro and in vivo models	Inhibits melanoma growth and cell migration	[86]
	SAMMSON-specific antisense oligonucleotide	Patient- derived xenograft	Induces apoptosis Exerts a synergistic anti-tumour effect with dabrafenib	[81]

## References

[1] Paluncic J., Kovacevic Z., Jansson P.J., Kalinowski D., Merlot A., Huang M.L.-H., Lok H., Sahni S., Lane D.J.R., Richardson D.R. (2016). Roads to melanoma: Key pathways and emerging players in melanoma progression and oncogenic signaling. Biochim. Biophys. Acta.

[2] Lazar A.D., Dinescu S., Costache M. (2020). Deciphering the Molecular Landscape of Cutaneous Squamous Cell Carcinoma for Better Diagnosis and Treatment. J. Clin. Med..

[3] Sommer L. (2011). Generation of melanocytes from neural crest cells. Pigment Cell Melanoma Res..

[4] Hussein M.R. (2005). Ultraviolet radiation and skin cancer: Molecular mechanisms. J. Cutan Pathol..

[5] Lopez A.T., Liu L., Geskin L., Blumenberg M. (2017). Molecular Mechanisms and Biomarkers of Skin Photocarcinogenesis. Human Skin Cancers: Pathways, Mechanisms, Targets and Treatments.

[6] Cancer Genome Atlas Network (2015). Genomic Classification of Cutaneous Melanoma. Cell.

[7] Clark W.H., Elder D.E., Guerry D.T., Epstein M.N., Greene M.H., Van Horn M. (1984). A study of tumor progression: The precursor lesions of superficial spreading and nodular melanoma. Hum. Pathol..

[8] Damsky W.E., Theodosakis N., Bosenberg M. (2014). Melanoma metastasis: New concepts and evolving paradigms. Oncogene.

[9] Hussussian C.J., Struewing J.P., Goldstein A.M., Higgins P.A., Ally D.S., Sheahan M.D., Clark W.H., Tucker M.A., Dracopoli N.C. (1994). Germline p16 mutations in familial melanoma. Nat. Genet..

[10] Sviderskaya E.V., Gray-Schopfer V.C., Hill S.P., Smit N.P., Evans-Whipp T.J., Bond J., Hill L., Bataille V., Peters G., Kipling D. (2003). p16/cyclin-dependent kinase inhibitor 2A deficiency in human melanocyte senescence, apoptosis, and immortalization: Possible implications for melanoma progression. J. Natl. Cancer Inst..

[11] Bennett D.C. (2003). Human melanocyte senescence and melanoma susceptibility genes. Oncogene.

[12] Miller A.J., Mihm M.C. (2006). Melanoma. N. Engl. J. Med..

[13] Shepelin D., Korzinkin M., Vanyushina A., Aliper A., Borisov N., Vasilov R., Zhukov N., Sokov D., Prassolov V., Gaifullin N. (2016). Molecular pathway activation features linked with transition from normal skin to primary and metastatic melanomas in human. Oncotarget.

[14] Turajlic S., Swanton C. (2016). Metastasis as an evolutionary process. Science.

[15] Lambert A.W., Pattabiraman D.R., Weinberg R.A. (2017). Emerging biological principles of metastasis. Cell.

[16] Klein C.A. (2013). Selection and adaptation during metastatic cancer progression. Nature.

[17] Stoecklein N.H., Klein C.A. (2010). Genetic disparity between primary tumours, disseminated tumour cells, and manifest metastasis. Int. J. Cancer.

[18] Eyles J., Puaux A.L., Wang X., Tan T.G., Zheng L., Ong L.C., Jin Y., Kato M., Prevost-Blondel A., Chow P. (2010). Tumor cells disseminate early, but immunosurveillance limits metastatic outgrowth, in a mouse model of melanoma. J. Clin. Investig..

[19] Klein C.A. (2009). Parallel progression of primary tumours and metastases. Nat. Rev. Cancer.

[20] Brastianos P.K., Carter S.L., Santagata S., Cahill D.P., Taylor-Weiner A., Jones R.T., Van Allen E.M., Lawrence M.S., Horowitz P.M., Cibulskis K. (2015). Genomic characterization of brain metastases reveals branched evolution and potential therapeutic targets. Cancer Discov..

[21] Werner-Klein M., Scheitler S., Hoffmann M., Hodak I., Dietz K., Lehnert P., Naimer V., Polzer B., Treitschke S., Werno C. (2018). Genetic alterations driving metastatic colony formation are acquired outside of the primary tumour in melanoma. Nat. Commun..

[22] Long G.V., Stroyakovskiy D., Gogas H., Levchenko E., de Braud F., Larkin J., Garbe C., Jouary T., Hauschild A., Grob J.J. (2014). Combined BRAF and MEK inhibition versus BRAF inhibition alone in melanoma. N. Engl. J. Med..

[23] Rizos H., Menzies A.M., Pupo G.M., Carlino M.S., Fung C., Hyman J., Haydu L.E., Mijatov B., Becker T.M., Boyd S.C. (2014). BRAF inhibitor resistance mechanisms in metastatic melanoma: Spectrum and clinical impact. Clin. Cancer Res..

[24] Gatzka M.V. (2018). Targeted Tumor Therapy Remixed-An Update on the Use of Small-Molecule Drugs in Combination Therapies. Cancers.

[25] Larkin J., Hodi F.S., Wolchok J.D. (2015). Combined Nivolumab and Ipilimumab or Monotherapy in Untreated Melanoma. N. Engl. J. Med..

[26] Robert C., Schachter J., Long G.V., Arance A., Grob J.J., Mortier L., Daud A., Carlino M.S., McNeil C., Lotem M. (2015). KEYNOTE-006 investigators. Pembrolizumab versus Ipilimumab in Advanced Melanoma. N. Engl. J. Med..

[27] Ribas A., Wolchok J.D. (2018). Cancer immunotherapy using checkpoint blockade. Science.

[28] Gajos-Michniewicz A., Czyz M. (2019). Role of miRNAs in Melanoma Metastasis. Cancers.

[29] Yu X., Zheng H., Tse G., Chan M.T.V., Wu W.K.K. (2018). Long non-coding RNAs in melanoma. Cell Prolif..

[30] Jarroux J., Morillon A., Pinskaya M. (2017). History, Discovery, and Classification of lncRNAs. Adv. Exp. Med. Biol..

[31] Luo C., Weber C.E.M., Osen W., Bosserhoff A.-K., Eichmüller S.B. (2014). The role of microRNAs in melanoma. Eur. J. Cell Biol..

[32] Hirota K., Miyoshi T., Kugou K., Hoffman C.S., Shibata T., Ohta K. (2008). Stepwise chromatin remodelling by a cascade of transcription initiation of non-coding RNAs. Nature.

[33] Brockdorff N. (2013). Noncoding RNA and Polycomb recruitment. RNA.

[34] Hansen T.B., Jensen T.I., Clausen B.H., Bramsen J.B., Finsen B., Damgaard C.K., Kjems J. (2013). Natural RNA circles function as efficient microRNA sponges. Nature.

[35] Segura M.F., Greenwald H.S., Hanniford D., Osman I., Hernando E. (2012). MicroRNA and cutaneous melanoma: From discovery to prognosis and therapy. Carcinogenesis.

[36] Mannavola F., Tucci M., Felici C., Stucci S., Silvestris F. (2016). miRNAs in melanoma: A defined role in tumor progression and metastasis. Exp. Rev. Clin. Immunol..

[37] Varrone F., Caputo E. (2020). The miRNAs Role in Melanoma and in Its Resistance to Therapy. Int. J. Mol. Sci..

[38] Garraway L.A., Widlund H.R., Rubin M.A., Getz G., Berger A.J., Ramaswamy S., Beroukhim R., Milner D.A., Granter S.R., Du J. (2005). Integrative genomic analyses identify MITF as a lineage survival oncogene amplified in malignant melanoma. Nature.

[39] Carreira S., Goodall J., Denat L., Rodriguez M., Nuciforo P., Hoek K.S., Testori A., Larue L., Goding C.R. (2006). Mitf regulation of Dia1 controls melanoma proliferation and invasiveness. Genes Dev..

[40] Bell R.E., Levy C. (2011). The three M’s: Melanoma, microphthalmia-associated transcription factor and microRNA. Pigment. Cell Melanoma Res..

[41] Bemis L.T., Chen R., Amato C.M., Classen E.H., Robinson S.E., Coffey D.G., Erickson P.F., Shellman G., Robinson W.A.F. (2008). MicroRNA-137 targets microphthalmia-associated transcription factor in melanoma cell lines. Cancer Res..

[42] Segura M.F., Hanniford D., Menendez S., Reavie L., Zou X., Alvarez-Diaz S., Zakrzewski J., Blochin E., Rose A., Bogunovic D. (2009). Aberrant miR-182 expression promotes melanoma metastasis by repressing FOXO3 and microphthalmia-associated transcription factor. Proc. Natl. Acad. Sci. USA.

[43] Boyle G.M., Woods S.L., Bonazzi V.F., Stark M.S., Hacker E., Aoude L.G., Dutton-Regester K., Cook A.L., Sturm R.A., Hayward N.K. (2011). Melanoma cell invasiveness is regulated by miR-211 suppression of the BRN2 transcription factor. Pigment Cell Melanoma Res..

[44] Zhao G., Wei Z., Guo Y. (2020). MicroRNA-107 is a novel tumor suppressor targeting POU3F2 in melanoma. Biol. Res..

[45] Luo C., Tetteh P.W., Merz P.R., Dickes E., Abukiwan A., Hotz-Wagenblatt A., Holland-Cunz S., Sinnberg T., Schittek B., Schadendorf D. (2013). MiR-137 inhibits the invasion of melanoma cells through downregulation of multiple oncogenic target genes. J. Investig. Derm..

[46] Mueller D.W., Rehli M., Bosserhoff A.K. (2009). MiRNA expression profiling in melanocytes and melanoma cell lines reveals miRNAs associated with formation and progression of malignant melanoma. J. Investig. Derm..

[47] Bell R.E., Khaled M., Netanely D., Schubert S., Golan T., Buxbaum A., Janas M.M., Postolsky B., Goldberg M.S., Shamir R. (2014). Transcription factor/microRNA axis blocks melanoma invasion program by miR-211 targeting NUAK1. J. Investig. Derm..

[48] Mazar J., DeYoung K., Khaitan D., Meister E., Almodovar A., Goydos J., Ray A., Perera R.J. (2010). The regulation of miRNA-211 expression and its role in melanoma cell invasiveness. PLoS ONE.

[49] Arozarena I., Sanchez-Laorden B., Packer L., Hidalgo-Carcedo C., Hayward R., Viros A., Sahai E., Marais R. (2011). Oncogenic BRAF induces melanoma cell invasion by downregulating the cGMP-specific phosphodiesterase PDE5A. Cancer Cell.

[50] Pan Z., Tian Y., Niu G., Cao C. (2020). Role of microRNAs in remodeling the tumor microenvironment (Review). Int. J. Oncol..

[51] Müller D.W., Bosserhoff A.K. (2008). Integrin beta 3 expression is regulated by let-7a miRNA in malignant melanoma. Oncogene.

[52] Fu T.Y., Chang C.C., Lin C.T., Lai C.H., Peng S.Y., Ko Y.J., Tang P.C. (2011). Let-7b-mediated suppression of basigin expression and metastasis in mouse melanoma cells. Exp. Cell Res..

[53] Martin del Campo S.E., Latchana N., Levine K.M., Grignol V.P., Fairchild E.T., Jaime-Ramirez A.C., Dao T.V., Karpa V.I., Carson M., Ganju A. (2015). MiR-21 enhances melanoma invasiveness via inhibition of tissue inhibitor of metalloproteinases 3 expression: In vivo effects of MiR-21 inhibitor. PLoS ONE.

[54] Gaziel-Sovran A., Segura M.F., Di Micco R., Collins M.K., Hanniford D., Vega-Saenz de Miera E., Rakus J.F., Dankert J.F., Shang S., Kerbel R.S. (2011). MiR30b/30d regulation of GalNAc transferases enhances invasion and immunosuppression during metastasis. Cancer Cell.

[55] Knoll S., Fürst K., Kowtharapu B., Schmitz U., Marquardt S., Wolkenhauer O., Martin H., Pützer B.M. (2014). E2F1 induces miR 224/452 expression to drive EMT through TXNIP downregulation. EMBO Rep..

[56] Zhong X., Zheng L., Shen J., Zhang D., Xiong M., Zhang Y., He X., Tanyi J.L., Yang F., Montone K.T. (2016). Suppression of MicroRNA 200 Family Expression by Oncogenic KRAS Activation Promotes Cell Survival and Epithelial-Mesenchymal Transition in KRAS-Driven Cancer. Mol. Cell. Biol..

[57] Penna E., Orso F., Cimino D., Tenaglia E., Lembo A., Quaglino E., Poliseno L., Haimovic A., Osella-Abate S., De Pittà C. (2011). microRNA-214 contributes to melanoma tumour progression through suppression of TFAP2C. EMBO J..

[58] Rambow F., Bechadergue A., Luciani F., Gros G., Domingues M., Bonaventure J., Meurice G., Marine J.C., Larue L. (2016). Regulation of Melanoma Progression through the TCF4/miR-125b/NEDD9 Cascade. J. Investig. Derm..

[59] Domingues M.J., Rambow F., Job B., Papon L., Liu W., Larue L., Bonaventure J. (2014). Beta-catenin inhibitor ICAT modulates the invasive motility of melanoma cells. Cancer Res..

[60] Pencheva N., Tran H., Buss C., Huh D., Drobnjak M., Busam K., Tavazoie S.F. (2012). Convergent multi-miRNA targeting of ApoE drives LRP1/LRP8 dependent melanoma metastasis and angiogenesis. Cell.

[61] Migliore C., Petrelli A., Ghiso E., Corso S., Capparuccia L., Eramo A., Comoglio P.M., Giordano S. (2008). MicroRNAs impair MET-mediated invasive growth. Cancer Res..

[62] Tucci M., Mannavola F., Passarelli A., Stucci L.S., Cives M., Silvestris F. (2018). Exosomes in melanoma: A role in tumor progression, metastasis and impaired immune system activity. Oncotarget.

[63] Gowda R., Robertson B.M., Iyer S., Barry J., Dinavahi S.S., Robertson G.P. (2020). The role of exosomes in metastasis and progression of melanoma. Cancer Treat. Rev..

[64] Hood J.L., San R.S., Wickline S.A. (2011). Exosomes released by melanoma cells prepare sentinel lymph nodes for tumor metastasis. Cancer Res..

[65] Hood J.L. (2016). Melanoma exosomes enable tumor tolerance in lymph nodes. Med. Hypotheses.

[66] Peinado H., Zhang H., Matei I.R., Costa-Silva B., Hoshino A., Rodrigues G., Psaila B., Kaplan R.N., Bromberg J.F., Kang Y. (2017). Pre-metastatic niches: Organ-specific homes for metastases. Nat. Rev. Cancer.

[67] Bhan A., Soleimani M., Mandal S.S. (2017). Long Noncoding RNA and Cancer: A New Paradigm. Cancer Res..

[68] Dinescu S., Ignat S., Lazar A.D., Constantin C., Neagu M., Costache M. (2019). Epitranscriptomic Signatures in lncRNAs and Their Possible Roles in Cancer. Genes.

[69] Dalay N. (2016). Role of the lncRNAs in malignant melanoma and their involvement in metastasis. Transl Cancer Res..

[70] Khaitan D., Dinger M.E., Mazar J., Crawford J., Smith M.A., Mattick J.S., Perera R.J. (2011). The melanoma-upregulated long noncoding RNA SPRY4-IT1 modulates apoptosis and invasion. Cancer Res..

[71] Mazar J., Zhao W., Khalil A.M., Lee B., Shelley J., Govindarajan S.S., Yamamoto F., Ratnam M., Aftab M.N., Collins S. (2014). The functional characterization of long noncoding RNA SPRY4-IT1 in human melanoma cells. Oncotarget.

[72] Sun M., Liu X.H., Lu K.H., Nie F.Q., Xia R., Kong R., Yang J.S., Xi T.P., Liu Y.W., Zou Y.F. (2014). EZH2-mediated epigenetic suppression of long noncoding RNA SPRY4-IT1 promotes NSCLC cell proliferation and metastasis by affecting the epithelial-mesenchymal transition. Cell Death Dis..

[73] Siena Á.D.D., Plaça J.R., Araújo L.F., Ichihara de Barros I., Peronni K., Molfetta G., Oliveira de Biagi C.A., Espreafico E.M., Sousa J.F., Silva W.A. (2019). Whole transcriptome analysis reveals correlation of long noncoding RNA ZEB1-AS1 with invasive profile in melanoma. Sci. Rep..

[74] Schmidt K., Joyce C.E., Buquicchio F., Brown A., Ritz J., Distel R.J., Yoon C.H., Novina C.D. (2016). The lncRNA SLNCR1 Mediates Melanoma Invasion through a Conserved SRA1-like Region. Cell Rep..

[75] Hombach S., Kretz M. (2013). The non-coding skin: Exploring the roles of long non-coding RNAs in epidermal homeostasis and disease. Bioessays.

[76] Flockhart R.J., Webster D.E., Qu K., Mascarenhas N., Kovalski J., Kretz M., Khavari P.A. (2012). BRAFV600E remodels the melanocyte transcriptome and induces BANCR to regulate melanoma cell migration. Genome Res..

[77] Akhbari P., Whitehouse A., Boyne J.R. (2014). Long non-coding RNAs drive metastatic progression in melanoma. Int J. Oncol..

[78] Pasmant E., Laurendeau I., Heron D., Vidaud M., Vidaud D., Bieche I. (2007). Characterization of a germ-line deletion, including the entire INK4/ARF locus, in a melanoma-neural system tumor family: Identification of ANRIL, an antisense noncoding RNA whose expression coclusters with ARF. Cancer Res..

[79] Xie H., Rachakonda P.S., Heidenreich B., Nagore E., Sucker A., Hemminki K., Schadendorf D., Kumar R. (2016). Mapping of deletion breakpoints at the CDKN2A locus in melanoma: Detection of MTAP-ANRIL fusion transcripts. Oncotarget.

[80] Xu S., Wang H., Pan H., Shi Y., Li T., Ge S., Jia R., Zhang H., Fan X. (2016). ANRIL lncRNA triggers efficient therapeutic efficacy by reprogramming the aberrant INK4-hub in melanoma. Cancer Lett..

[81] Leucci E., Vendramin R., Spinazzi M., Laurette P., Fiers M., Wouters J., Radaelli E., Eyckerman S., Leonelli C., Vanderheyden K. (2016). Melanoma addiction to the long non-coding RNA SAMMSON. Nature.

[82] Tang L., Zhang W., Su B., Yu B. (2013). Long noncoding RNA HOTAIR is associated with motility, invasion, and metastatic potential of metastatic melanoma. Biomed. Res. Int..

[83] Tian Y., Zhang X., Hao Y., Fang Z., He Y. (2014). Potential roles of abnormally expressed long noncoding RNA UCA1 and Malat-1 in metastasis of melanoma. Melanoma Res..

[84] Wei Y., Sun Q., Zhao L., Wu J., Chen X., Wang Y., Zang W., Zhao G. (2016). LncRNA UCA1-miR-507-FOXM1 axis is involved in cell proliferation, invasion and G0/G1 cell cycle arrest in melanoma. Med. Oncol..

[85] Pan B.M., Lin X., Zhang L., Hong W., Zhang Y. (2019). Long noncoding RNA X-inactive specific transcript promotes malignant melanoma progression and oxaliplatin resistance. Melanoma Res..

[86] Chen L., Yang H., Xiao Y., Tang X., Li Y., Han Q., Fu J., Yang Y., Zhu Y. (2016). LncRNA GAS5 is a critical regulator of metastasis phenotype of melanoma cells and inhibits tumor growth in vivo. OncoTargets.

[87] Wu L., Zhu L., Li Y., Zheng Z., Lin X., Yang C. (2020). LncRNA MEG3 promotes melanoma growth, metastasis and formation through modulating miR-21/E-cadherin axis. Cancer Cell Int..

[88] Chen L., Yang H., Xiao Y., Tang X., Li Y., Han Q., Fu J., Yang Y., Zhu Y. (2016). Lentiviral-mediated overexpression of long noncoding RNA GAS5 reduces invasion by mediating MMP2 expression and activity in human melanoma cells. Int J. Oncol..

[89] Bian D., Shi W., Shao Y., Li P., Song G. (2017). Long non-coding RNA GAS5 inhibits tumorigenesis via miR-137 in melanoma. Am. J. Transl. Res..

[90] Lorusso C., De Summa S., Pinto R., Danza K., Tommasi S. (2020). miRNAs as Key Players in the Management of Cutaneous Melanoma. Cells.

[91] Dror S., Sander L., Schwartz H., Sheinboim D., Barzilai A., Dishon Y., Apcher S., Golan T., Greenberger S., Barshack I. (2016). Melanoma miRNA trafficking controls tumour primary niche formation. Nat. Cell Biol..

[92] Hanna S.C., Krishnan B., Bailey S.T., Moschos S.J., Kuan P.F., Shimamura T., Osborne L.D., Siegel M.B., Duncan L.M., O’Brien E.T. (2013). HIF1α and HIF2α independently activate SRC to promote melanoma metastases. J. Clin. Investig..

[93] King H.W., Michael M.Z., Gleadle J.M. (2012). Hypoxic enhancement of exosome release by breast cancer cells. BMC Cancer.

[94] Wozniak M., Peczek L., Czernek L., Düchler M. (2017). Analysis of the miRNA profiles of melanoma exosomes derived under normoxic and hypoxic culture conditions. Anticancer Res..

[95] Majmundar A.J., Wong W.J., Simon M.C. (2010). Hypoxia-inducible factors and the response to hypoxic stress. Mol. Cell.

[96] Hwang H.W., Baxter L.L., Loftus S.K., Cronin J.C., Trivedi N.S., Borate B., Pavan W.J. (2014). Distinct microRNA expression signatures are associated with melanoma subtypes and are regulated by HIF1A. Pigment Cell Melanoma Res..

[97] Maes H., Van Eygen S., Krysko D.V., Vandenabeele P., Nys K., Rillaerts K., Garg A.D., Verfaillie T., Agostinis P. (2014). BNIP3 supports melanoma cell migration and vasculogenic mimicry by orchestrating the actin cytoskeleton. Cell Death Dis..

[98] Vara-Perez M., Maes H., Van Dingenen S., Agostinis P. (2019). BNIP3 contributes to the glutamine-driven aggressive behavior of melanoma cells. Biol. Chem..

[99] Mazar J., Qi F., Lee B., Marchica J., Govindarajan S., Shelley J., Li J.-L., Ray A., Perera R.J. (2016). MicroRNA 211 Functions as a Metabolic Switch in Human Melanoma Cells. Mol. Cell. Biol..

[100] Chung A.S., Lee J., Ferrara N. (2010). Targeting the tumour vasculature: Insights from physiological angiogenesis. Nat. Rev. Cancer.

[101] Bergers G., Benjamin L.E. (2003). Tumorigenesis and the angiogenic switch. Nat. Rev. Cancer.

[102] Zhuang G., Wu X., Jiang Z., Kasman I., Yao J., Guan Y., Oeh J., Modrusan Z., Bais C., Sampath D. (2012). Tumour-secreted miR-9 promotes endothelial cell migration and angiogenesis by activating the JAK-STAT pathway. EMBO J..

[103] Crawford Y., Kasman I., Yu L., Zhong C., Wu X., Modrusan Z., Kaminker J., Ferrara N. (2009). PDGF-C mediates the angiogenic and tumorigenic properties of fibroblasts associated with tumors refractory to anti-VEGF treatment. Cancer Cell.

[104] Anderberg C., Li H., Fredriksson L., Andrae J., Betsholtz C., Li X., Eriksson U., Pietras K. (2009). Paracrine signaling by platelet-derived growth factor-CC promotes tumor growth by recruitment of cancer-associated fibroblasts. Cancer Res..

[105] Vallacchi V., Camisaschi C., Dugo M., Vergani E., Deho P., Gualeni A., Huber V., Gloghini A., Maurichi A., Santinami M. (2016). MicroRNA Expression in Sentinel Nodes from Progressing Melanoma Patients Identifies Networks Associated with Dysfunctional Immune Response. Genes.

[106] Noman M.Z., Buart S., Romero P., Ketari S., Janji B., Mari B., Mami-Chouaib F., Chouaib S. (2012). Hypoxia-inducible miR-210 regulates the susceptibility of tumor cells to lysis by cytotoxic T cells. Cancer Res..

[107] Chen S., Wang L., Fan J., Ye C., Dominguez D., Zhang Y., Curiel T.J., Fang D., Kuzel T.M., Zhang B. (2015). Host miR155 promotes tumor growth through a myeloid-derived suppressor cell-dependent mechanism. Cancer Res..

[108] Valenti R., Huber V., Iero M., Filipazzi P., Parmiani G., Rivoltini L. (2007). Tumor-released microvesicles as vehicles of immunosuppression. Cancer Res..

[109] Ding H., Yang X., Wei Y. (2018). Fusion Proteins of NKG2D/NKG2DL in Cancer Immunotherapy. Int. J. Mol. Sci..

[110] Heinemann A., Zhao F., Pechlivanis S., Eberle J., Steinle A., Diederichs S., Schadendorf D., Paschen A. (2012). Tumor suppressive microRNAs miR-34a/c control cancer cell expression of ULBP2, a stress-induced ligand of the natural killer cell receptor NKG2D. Cancer Res..

[111] Paschen A., Sucker A., Hill B., Moll I., Zapatka M., Nguyen X.D., Sim G.C., Gutmann I., Hassel J., Becker J.C. (2009). Differential clinical significance of individual NKG2D ligands in melanoma: Soluble ULBP2 as an indicator of poor prognosis superior to S100B. Clin. Cancer Res..

[112] Pucci F., Garris C., Lai C.P., Newton A., Pfirschke C., Engblom C., Alvarez D., Sprachman M., Evavold C., Magnuson A. (2016). SCS macrophages suppress melanoma by restricting tumor-derived vesicle-B cell interactions. Science.

[113] Li K., Chen Y., Li A., Tan C., Liu X. (2019). Exosomes Play Roles in Sequential Processes of Tumor Metastasis. Int J. Cancer.

[114] Cantile M., Scognamiglio G., Marra L., Aquino G., Botti C., Falcone M., Malzone M.G., Liguori G., Di Bonito M., Franco R. (2017). HOTAIR role in melanoma progression and its identification in the blood of patients with advanced disease. J. Cell Physiol..

[115] Obaid M., Udden S.M.N., Deb P., Shihabeddin N., Zaki M.H., Mandal S.S. (2018). LncRNA HOTAIR regulates lipopolysaccharide-induced cytokine expression and inflammatory response in macrophages. Sci. Rep..

[116] Botti G., Scognamiglio G., Aquino G., Liguori G., Cantile M. (2019). LncRNA HOTAIR in Tumor Microenvironment: What Role?. Int. J. Mol. Sci..

[117] Vergani E., Di Guardo L., Dugo M., Rigoletto S., Tragni G., Ruggeri R., Perrone F., Tamborini E., Gloghini A., Arienti F. (2016). Overcoming melanoma resistance to vemurafenib by targeting CCL2-induced miR-34a, miR-100 and miR-125b. Oncotarget.

[118] Koetz-Ploch L., Hanniford D., Dolgalev I., Sokolova E., Zhong J., Diaz-Martinez M., Bernstein E., Darvishian F., Flaherty K.T., Chapman P.B. (2017). MicroRNA-125a promotes resistance to BRAF inhibitors through suppression of the intrinsic apoptotic pathway. Pigment Cell Melanoma Res..

[119] Díaz-Martínez M., Benito-Jardón L., Alonso L., Koetz-Ploch L., Hernando E., Teixidó J. (2018). MiR-204-5p and miR-211-5p Contribute to BRAF Inhibitor Resistance in Melanoma. Cancer Res..

[120] Vitiello M., D’Aurizio R., Poliseno L. (2018). Biological role of miR-204 and miR-211 in melanoma. Oncoscience.

[121] Stark M.S., Bonazzi V.F., Boyle G.M., Palmer J.M., Symmons J., Lanagan C.M., Schmidt C.W., Herington A.C., Ballotti R., Pollock P.M. (2015). miR-514a regulates the tumour suppressor NF1 and modulates BRAFi sensitivity in melanoma. Oncotarget.

[122] Sun X., Li J., Sun Y., Zhang Y., Dong L., Shen C., Yang L., Yang M., Li Y., Shen G. (2016). miR-7 reverses the resistance to BRAFi in melanoma by targeting EGFR/IGF-1R/CRAF and inhibiting the MAPK and PI3K/AKT signaling pathways. Oncotarget.

[123] Mishra P.J., Merlino G. (2016). Integrated Genomics Identifies miR-32/MCL-1 Pathway as a Critical Driver of Melanomagenesis: Implications for miR-Replacement and Combination Therapy. PLoS ONE.

[124] Liu S., Tetzlaff M.T., Wang T., Yang R., Xie L., Zhang G., Krepler C., Xiao M., Beqiri M., Xu W. (2015). miR-200c/Bmi1 axis and epithelial-mesenchymal transition contribute to acquired resistance to BRAF inhibitor treatment. Pigment Cell Melanoma Res..

[125] Fattore L., Mancini R., Acunzo M., Romano G., Lagana A., Pisanu M.E., Malpicci D., Madonna G., Mallardo D., Capone M. (2016). miR-579-3p controls melanoma progression and resistance to target therapy. Proc. Natl. Acad. Sci. USA.

[126] Sanlorenzo M., Vujic I., Esteve-Puig R., Lai K., Vujic M., Lin K., Posch C., Dimon M., Moy A., Zekhtser M. (2018). The lincRNA MIRAT binds to IQGAP1 and modulates the MAPK pathway in NRAS mutant melanoma. Sci. Rep..

[127] Joung J., Engreitz J.M., Konermann S., Abudayyeh O.O., Verdine V.K., Aguet F. (2017). Genome-scale activation screen identifies a lncRNA locus regulating a gene neighbourhood. Nature.

[128] Vishnubalaji R., Hibah S., Elango R., Alajez N.M. (2020). Noncoding RNAs as potential mediators of resistance to cancer immunotherapy. Semin. Cancer Biol..

[129] Zhou Y., Zhu Y., Xie Y., Ma X. (2019). The Role of Long Non-coding RNAs in Immunotherapy Resistance. Front. Oncol..

[130] Huber V., Vallacchi V., Fleming V., Hu X., Cova A., Dugo M., Shahaj E., Sulsenti R., Vergani E., Filipazzi P. (2018). Tumor-derived microRNAs induce myeloid suppressor cells and predict immunotherapy resistance in melanoma. J. Clin. Investig..

[131] Li Q., Johnston N., Zheng X., Wang H., Zhang X., Gao D., Min W. (2016). miR-28 modulates exhaustive differentiation of T cells through silencing programmed cell death-1 and regulating cytokine secretion. Oncotarget.

[132] Audrito V., Serra S., Stingi A., Orso F., Gaudino F., Bologna C., Neri F., Garaffo G., Nassini R., Baroni G. (2017). PD-L1 up-regulation in melanoma increases disease aggressiveness and is mediated through miR-17-5p. Oncotarget.

[133] Shang W., Gao Y., Tang Z., Zhang Y., Yang R. (2019). The pseudogene Olfr29-ps1 promotes the suppressive function and differentiation of monocytic MDSCs. Cancer Immunol. Res..

[134] Gao Y., Wang T., Li Y., Zhang Y., Yang R. (2018). Lnc-chop promotes immunosuppressive function of myeloid-derived suppressor cells in tumor and inflammatory environments. J. Immunol..

[135] Charpentier M., Croyal M., Carbonnelle D., Fortun A., Florenceau L., Rabu C., Krempf M., Labarriere N., Lang F. (2016). IRES-dependent translation of the long non coding RNA meloe in melanoma cells produces the most immunogenic MELOE antigens. Oncotarget.

[136] Godet Y., Moreau-Aubry A., Guilloux Y., Vignard V., Khammari A., Dreno B., Jotereau F., Labarriere N. (2008). MELOE-1 is a new antigen overexpressed in melanomas and involved in adoptive T cell transfer efficiency. J. Exp. Med..

[137] Palmirotta R., Lovero D., Cafforio P., Felici C., Mannavola F., Pellè E., Quaresmini D., Tucci M., Silvestris F. (2018). Liquid biopsy of cancer: A multimodal diagnostic tool in clinical oncology. Adv. Med. Oncol..

[138] Schwarzenbach H., Hoon D.S.B., Pantel K. (2011). Cell-free nucleic acids as biomarkers in cancer patients. Nat. Rev. Cancer.

[139] Sole C., Arnaiz E., Manterola L., Otaegui D., Lawrie C.H. (2019). The circulating transcriptome as a source of cancer liquid biopsy biomarkers. Semin. Cancer Biol..

[140] Leidinger P., Keller A., Borries A., Reichrath J., Rass K., Jager S.V., Lenhof H.-P., Meeseet E. (2010). High-throughput miRNA profiling of human melanoma blood samples. BMC Cancer.

[141] Mumford S.L., Towler B.P., Pashler A.L., Gilleard O., Martin Y., Newbury S.F. (2018). Circulating MicroRNA Biomarkers in Melanoma: Tools and Challenges in Personalised Medicine. Biomolecules.

[142] Pfeffer S.R., Grossmann K.F., Cassidy P.B., Yang C.H., Fan M., Kopelovich L., Leachman S.A., Pfeffer L.M. (2015). Detection of Exosomal miRNAs in the Plasma of Melanoma Patients. J. Clin. Med..

[143] Mannavola F., D’Oronzo S., Cives M., Stucci L.S., Ranieri G., Silvestris F., Tucci M. (2020). Extracellular Vesicles and Epigenetic Modifications Are Hallmarks of Melanoma Progression. Int. J. Mol. Sci..

[144] Armand-Labit V., Meyer N., Casanova A., Bonnabau H., Platzer V., Tournier E., Sansas B., Verdun S., Thouvenot B., Hilselberger B. (2016). Identification of a Circulating MicroRNA Profile as a Biomarker of Metastatic Cutaneous Melanoma. Acta Derm. Venereol..

[145] Van Laar R., Lincoln M., Van Laar B. (2018). Development and validation of a plasma-based melanoma biomarker suitable for clinical use. Br. J. Cancer.

[146] Li P., He Q.Y., Luo C.Q., Qian L.Y. (2014). Circulating miR-221 expression level and prognosis of cutaneous malignant melanoma. Med. Sci Monit..

[147] Fleming N.H., Zhong J., Da Silva I.P., De Miera E.V.S., Brady B., Han S.W., Hanniford D., Wang J., Shapiro R.L., Hernando E. (2015). Serum-based miRNAs in the prediction and detection of recurrence in melanoma patients. Cancer.

[148] Stark M.S., Klein K., Weide B., Haydu L.E., Pflugfelder A., Tang Y.H., Palmer J.M., Whiteman D.C., Scolyer R.A., Mann G.J. (2015). The Prognostic and Predictive Value of Melanoma-related MicroRNAs Using Tissue and Serum: A MicroRNA Expression Analysis. EBioMedicine.

[149] Liu T., Shen S.K., Xiong J.G., Xu Y., Zhang H.Q., Liu H.J., Lu Z.G. (2016). Clinical significance of long noncoding RNA SPRY4-IT1 in melanoma patients. FEBS Open Bio.

[150] Esteller M. (2011). Non-coding RNAs in human disease. Nat. Rev. Genet..

[151] Hulstaert E., Brochez L., Volders P.J., Vandesompele J., Mestdagh P. (2017). Long non-coding RNAs in cutaneous melanoma: Clinical perspectives. Oncotarget.

[152] Stenvang J., Petri A., Lindow M., Obad S., Kauppinen S. (2012). Inhibition of microRNA function by antimiR oligonucleotides. Silence.

[153] Hosseinahli N., Aghapour M., Duijf P.H.G., Baradaran B. (2018). Treating cancer with microRNA replacement therapy: A literature review. J. Cell Physiol..

[154] Fattore L., Campani V., Ruggiero C.F., Salvati V., Liguoro D., Scotti L., Botti G., Ascierto P.A., Mancini R., De Rosa G. (2020). In Vitro Biophysical and Biological Characterization of Lipid Nanoparticles Co-Encapsulating Oncosuppressors miR-199b-5p and miR-204-5p as Potentiators of Target Therapy in Metastatic Melanoma. Int. J. Molec. Sci..

[155] Aftab M.N., Dinger M.E., Perera R.J. (2014). The role of microRNAs and long non-coding RNAs in the pathology, diagnosis, and management of melanoma. Arch. Biochem. Biophys..

